# Combining environmental DNA and remote sensing for efficient, fine-scale mapping of arthropod biodiversity

**DOI:** 10.1098/rstb.2023.0123

**Published:** 2024-06-24

**Authors:** Yuanheng Li, Christian Devenish, Marie I. Tosa, Mingjie Luo, David M. Bell, Damon B. Lesmeister, Paul Greenfield, Maximilian Pichler, Taal Levi, Douglas W. Yu

**Affiliations:** ^1^ Yunnan Key Laboratory of Biodiversity and Ecological Security of Gaoligong Mountain, State Key Laboratory of Genetic Resources and Evolution, Chinese Academy of Sciences, Kunming, Yunnan 650223, People’s Republic of China; ^2^ Kunming Institute of Zoology, Chinese Academy of Sciences, Kunming, Yunnan 650223, People’s Republic of China; ^3^ Faculty of Biology, University of Duisburg-Essen, Essen 45141, Germany; ^4^ School of Biological Sciences, University of East Anglia, Norwich Research Park, Norwich, Norfolk NR47TJ, UK; ^5^ Department of Fisheries, Wildlife, and Conservation Sciences, Oregon State University, Corvallis, OR 97331, USA; ^6^ Kunming College of Life Sciences, University of Chinese Academy of Sciences, Kunming, People’s Republic of China; ^7^ Pacific Northwest Research Station, U.S. Department of Agriculture Forest Service, Corvallis, OR 97331, USA; ^8^ CSIRO Energy, Lindfield, New South Wales, Australia; ^9^ School of Biological Sciences, Macquarie University, Sydney, Australia; ^10^ Theoretical Ecology, University of Regensburg, Regensburg, Germany; ^11^ Center for Excellence in Animal Evolution and Genetics, Chinese Academy of Sciences, Kunming Yunnan 650223, People’s Republic of China

**Keywords:** environmental DNA, Earth observation, biodiversity indices, systematic conservation planning, forestry, machine learning

## Abstract

Arthropods contribute importantly to ecosystem functioning but remain understudied. This undermines the validity of conservation decisions. Modern methods are now making arthropods easier to study, since arthropods can be mass-trapped, mass-identified, and semi-mass-quantified into ‘many-row (observation), many-column (species)‘ datasets, with homogeneous error, high resolution, and copious environmental-covariate information. These ‘novel community datasets’ let us efficiently generate information on arthropod species distributions, conservation values, uncertainty, and the magnitude and direction of human impacts. We use a DNA-based method (barcode mapping) to produce an arthropod-community dataset from 121 Malaise-trap samples, and combine it with 29 remote-imagery layers using a deep neural net in a joint species distribution model. With this approach, we generate distribution maps for 76 arthropod species across a 225 km^2^ temperate-zone forested landscape. We combine the maps to visualize the fine-scale spatial distributions of species richness, community composition, and site irreplaceability. Old-growth forests show distinct community composition and higher species richness, and stream courses have the highest site-irreplaceability values. With this ‘sideways biodiversity modelling’ method, we demonstrate the feasibility of biodiversity mapping at sufficient spatial resolution to inform local management choices, while also being efficient enough to scale up to thousands of square kilometres.

This article is part of the theme issue ‘Towards a toolkit for global insect biodiversity monitoring’.

## 1. Introduction

Arthropods contribute in numerous ways to ecosystem functioning [1] but are understudied relative to vertebrates and plants [2]. This taxonomic bias undermines the validity of conservation decisions when the effects of change in climate, land use and land cover differ across taxa [3,4]. Also, it is arguable that modern methods now make arthropods *easier* to study than vertebrates and plants, given that arthropods can be mass-trapped and mass-identified [5,6]. Another logistical advantage is that arthropod community structure is correlated with vegetation structure [7,8], and since vegetation can be measured remotely at large spatial scale via airborne and spaceborne sensors [9], remote imagery could also provide large-spatial-scale information on arthropods. In fact, it is already known that spaceborne synthetic aperture radar, and airborne light detection and ranging (LiDAR) imagery of fine-scale forest structure can predict the distributions of entomofauna and avifauna [10–13].

### (a) Successful governance of the biodiversity commons

Arthropod conservation should be seen in the wider context of efficient biodiversity governance. Dietz *et al.*’s [14] framework for the successful governance of public goods can be usefully summarized into five elements: (i) information generation, (ii) infrastructure provision, (iii) political bargaining, (iv) enforcement and (v) institutional redesign. The first element, information generation, asks engineers and scientists to generate *high-quality*, *granular*, *timely*, *trustworthy* and *understandable* information on ecosystem status and change, values, uncertainty, and the magnitude and direction of human impacts.

Although there exists an example of the five elements working together to achieve single-species conservation (see the electronic supplementary material: ‘Dietz *et al.*’s five elements’), to our knowledge, there is so far no example of the five elements comprehensively working together to achieve *multi-species* conservation, in large part because the tools, study designs and analyses needed to generate information on many species at once are complex. This complexity is a barrier to uptake, delaying the institutional redesigns that could operationalize, finance and scale-up conservation.

Our focus in this study is therefore to demonstrate how to efficiently generate *high-quality*, *granular*, *timely*, *trustworthy* and *understandable* information on status and change in arthropod biodiversity, conservation value, uncertainty, and the magnitude and direction of human impacts.

We use the management of national forests in the United States (US) as our test case for multi-species biodiversity conservation. This management should follow the doctrine outlined in the 1960 Multiple-Use Sustained-Yield Act that requires management and use of natural resources to satisfy multiple competing interests and to maintain the natural resources in perpetuity [15–17]. Although US law mandates that each use be given equal priority, implementation is stymied by a lack of biodiversity data such as distribution maps of large numbers of species to identify areas of high conservation value that can be protected while still supporting extractive uses in other areas. Moreover, the species distribution maps should be regularly updated so that the impacts of management interventions can be inferred, feeding back to adaptive management [9,18].

### (b) High-throughput arthropod inventories

Now though, there are new technologies capable of efficiently and granularly capturing biodiversity information, via DNA isolated from environmental samples (eDNA) and via electronic sensors (bioacoustics, cameras, radar) [5,6,9,19–24]. The eDNA methods start with DNA-based taxonomic assignment (‘DNA barcoding’ [25]) and vary in how the DNA is collected and processed. For instance, large numbers of arthropods can efficiently be individually DNA-extracted and sequenced to produce count datasets [26,27]. These DNA-barcoded specimens (plus human-identified specimens) can optionally be used to annotate specimen images to train deep-learning models to scale up identifications [5,6]. Alternatively, DNA from arthropods can be extracted *en masse* from traps [28] or from environmental substrates, such as water washes of flowers (e.g. [29]) and mass-sequenced. These latter processing pipelines are known as ‘metabarcoding’ or ‘metagenomics’, depending on whether the target DNA-barcode sequence is polymerase chain reaction-amplified (both described in [9]).

The eDNA- and sensor-based methods can all produce ‘novel community data’, which Hartig *et al.* [30] describe as ‘many-row (observation), many-column (species)’ datasets, therefore making possible high spatial and/or temporal resolution and extent. Novel community data contain some form of abundance information, ranging from counts to within-species abundance change [31,32] to presence/absence, and because the methods are automated and standardized, the errors in these datasets tend to be homogeneous (e.g. minimal observer effects), which facilitates their correction given appropriate sample replicates and statistical models.

### (c) ‘Sideways’ biodiversity modelling and site irreplaceability ranking

It is natural to think about combining novel community data with copious environmental-covariate information in the form of continuous-space remote-imagery layers (and/or with continuous-time acoustic series) to produce continuous spatio(-temporal) biodiversity data products [9,30,33–40]. Here, we do just this, combining a point-sample dataset of Malaise-trapped arthropods with continuous-space Landsat and LiDAR imagery within a joint species distribution model (JSDM [40–43]). We were able to produce distribution maps for 76 arthropod species across a forested landscape. Because this landscape is characterized by overlapping gradients of environmental conditions (e.g. elevation, distance from streams and roads) and mosaics of management (e.g. clearcuts, old-growth), we can estimate the effects of different combinations of natural and anthropogenic drivers on arthropod biodiversity, including combinations that were not included in our sample set. We can also subdivide the landscape into management units and rank them by conservation value, to inform decision-making in this multi-use landscape.

The above approach is a direct test of a protocol originally proposed by Bush *et al.* [9] and more formally described by Pollock *et al.* [44] under the name ‘sideways’ biodiversity modelling. In short, sideways biodiversity models (i) integrate ‘the largely independent fields of biodiversity modelling and conservation’ [44, p. 1119] and (ii) include large numbers of species in conservation planning instead of using habitat-based metrics. Or in plain language, we use remote-sensing imagery to fill in the blanks between our sampling points, which creates a continuous map of arthropod biodiversity that we can use to study arthropod ecology and guide conservation.

## 2. Material and methods

In short, we combine DNA-based species detections, remote-sensing-derived environmental predictors, and joint species distribution modelling to predict and visualize the fine-scale distribution of arthropods across a large forested landscape. We use the joint predictions from the JSDM to map species richness, compositional distinctiveness and conservation value across the landscape. For the detailed protocol and explanations of the field, laboratory, bioinformatic and statistical methods, see electronic supplementary material: Materials and Methods.

### (a) Model Inputs

#### (i) Field data collection

**Figure 1.**
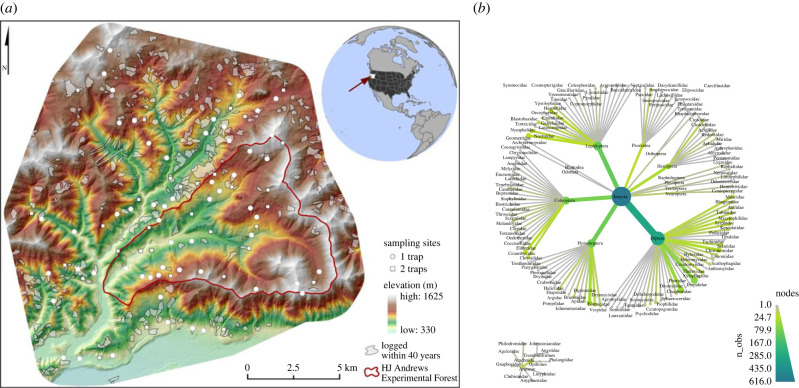
Sampling design and taxonomic diversity of the Malaise trapping campaign. (*a*) Sampling points in and around the H.J. Andrews Experimental Forest (red line), OR, USA. The study area consists of old-growth and logged (grey patches) deciduous and evergreen forest under different management regimes. Arthropods were sampled with Malaise traps at 89 sampling points in July 2018, with one trap at 57 points (white circles) and with two traps 40 m apart at 32 points (white squares). Elevation indicated with a green to white false-colour gradient. (*b*) Taxonomic distribution of all detected operational taxonomic units (OTUs) from the samples. Node size and colour are scaled to the number of OTUs. See the electronic supplementary material, figure S4 for a heat tree of the 190 included OTUs.

We collected with 121 Malaise-trap samples for seven days into 100% ethanol at 89 sampling points in and around the H.J. Andrews Experimental Forest (HJA), OR, USA in July 2018 (figure 1). Sites were stratified by elevation, time since disturbance, and inside and outside the HJA (inside, a long-term research site with no logging since 1989; outside, continued active management). HJA represents a range of previously logged to primary forest, but with notably larger areas of mature and old-growth forest reserves than the regional forest mosaic, which consists of short-rotation plantation forests on private land and a recent history of active management on public land.

#### (ii) Wet-laboratory pipeline and bioinformatics

#### (iii) DNA extraction and sequencing

We extracted the DNA from each Malaise-trap sample by soaking the arthropods in a lysis buffer and sent it to Novogene (Beijing, China) for whole-genome shotgun sequencing.

#### (iv) Creating a barcode reference database using Kelpie *in silico* polymerase chain reaction

On the output fastq files, we carried out ‘in silico’ PCR using Kelpie 2.0.11 [45] and the BF3 + BR2 primers from [46], outputting 5560 unique DNA-barcode sequences. After 97%-similarity clustering and filtering for erroneous sequences, we were left with 1225 operational taxonomic units (OTUs) as the reference barcode set.

#### (v) Read mapping to reference barcodes

We then mapped the reads of each sample to the reference barcodes, creating a 121 − sample × 1225 − OTU table. A species was accepted as being in a sample if reads mapped at high quality along more than 50% of its barcode length, following acceptance criteria from Ji *et al.* [47].

#### (vi) Environmental covariates

To predict species occurrences in the areas between the sampling points, we collected 58 continuous-space predictors (electronic supplementary material, table S1), relating to forest structure, vegetation reflectance and phenology, topography, and anthropogenic features, restricting ourselves to predictors that can be measured remotely. The forest-structure variables were from airborne LiDAR data collected from 2008 to 2016, which correlate with forest structure in US Pacific northwest coniferous forests, such as mean diameter, canopy cover and tree density [48]. The vegetation-related variables came from Landsat 8 individual bands, plus standard deviation, median, 5% and 95% percentiles of those bands over the year, and indices of vegetation status, e.g. normalized difference vegetation index. Both the proportion of canopy cover and annual Landsat metrics were calculated within radii of 100, 250 and 500 m, given that vegetation structure at different spatial scales is known to drive arthropod biodiversity [49]. The topography variables were calculated from LiDAR ground returns, including elevation, slope, eastness and northness split from aspect, topographic position index, topographic roughness index (TRI) [50], topographic wetness index [51] and distance to streams, based on a vector stream network (http://oregonexplorer.info, accessed 24 October 2019). The anthropogenic variables include distance to nearest road, proportion of area logged within the last 100 and within the last 40 years, within radii of 250, 500 and 1000 m, and a categorical variable of inside or outside the boundary of the HJA. They are not directly derived from remote-sensing data, but we included them because they could be derived from remote-sensing imagery. We then reduced our 58 environmental covariates to 29, removing the covariates that were most correlated with the others (as measured by variance inflation factor). The 29 retained covariates include six anthropogenic activities, two raw Landsat bands, seven indices based on annual Landsat data, six canopy/vegetation-related variables from LiDAR, and eight topography variables (electronic supplementary material, table S1 and figure S5), which we mapped across the study area at 30 m resolution.

### (b) Statistical analyses

#### (i) Species inputs

We converted the sample × species table to presence-absence data (1/0), and we only included species present at six or more sampling sites across the 121 samples. Our species dataset was thus reduced to 190 species in two classes, Insecta and Arachnida (figure 1*b*).

#### (ii) Joint species distribution model

The general idea behind species distribution modelling is to ‘predict a species’ distribution’. We use each species’ observed incidences (1/0) at all sampling points, plus the environmental-covariate values at those points, to ‘fit’ a model that predicts the species’ incidences from the covariate values. Once we have a fitted model, we use it to predict the species’ probability of presence over the rest of the sampling area, where the environmental-covariate values are known but the species’ incidences are not. Spatial autocorrelation was accounted by a trend-surface component. JSDMs extend individual species distribution models by additionally accounting for co-occurrences of species (see the electronic supplementary material: Joint Species Distribution Model).

#### (iii) Tuning and testing

The statistical challenge is to avoid overfitting, which is when the fitted model does a good job of predicting the species’ incidences at the sampling points that were used to fit the model in the first place but does a bad job of predicting the species over the rest of the landscape. Overfitting is likely in our dataset because many of our species are rare, there are many candidate remote-sensing covariates, and we expect that any relationships between remote-sensing-derived covariates and arthropod incidences are indirect and thus complex, necessitating the use of flexible mathematical functions.

To minimize overfitting, we used regularization and cross-validation. Regularization uses penalty terms during model fitting to favour a relatively simple set of covariates, and cross-validation finds the best values for those penalty terms (tuning). First, we randomly split the species incidence data from the 121 samples in 89 sampling points into 75% training data (*n* = 91) and 25% test data (*n* = 30) (electronic supplementary material, figure S1). The training data were used to try 1000 different hyperparameter combinations in a fivefold cross-validation design, some of which are the penalty terms, to find the combination that achieves the highest predictive performance on the training data itself (see the electronic supplementary material: Tuning and Testing, figure S1). The model with this combination was then applied to the 25% test data to measure true predictive performance. To fit the model, we used the JSDM R package sjSDM 1.0.5 [42], with the DNN deep neural network (DNN) option to account for complex, nonlinear effects of environmental covariates (the DNN outperformed a linear model; see the electronic supplementary material, figure S11), which suits our dataset of many species with few data points and many covariates.

Finally, to estimate how OTU incidence affects the variability of predictive accuracies, we also tuned a model to the whole dataset in a fivefold cross-validation, found optimal hyperparameters, and used them in another fivefold cross-validation on the entire dataset to estimate the variability of predictive area under the curve (AUCs) by OTU (see the electronic supplementary material: Variability in Predictive AUC by OTU Incidence). We emphasize that method is only useful for estimating variability in predictive performance, given that it potentially overestimates predictive performance, which is what we avoided by using a pure holdout in the main analysis.

#### (iv) Variable importance with explainable-artificial intelligence

The mathematical functions used in neural network models are unknown, but it would be useful to identify the covariates that contribute the most to explaining each species incidences. We therefore carried out an ‘explainable-artificial intelligence‘ (xAI) analysis, using the R package flashlight 0.8.0 [52]. In short, for each environmental-covariate, we shuffled its values in the dataset and estimated the drop in explanatory performance on the training data. The most important covariate is the one that, when permuted, degrades explanatory performance the most (see the electronic supplementary material: Variable importance with explainable AI (xAI)).

#### (v) Prediction and visualization of species distributions

Finally, after applying the final model to the test dataset, we identified 76 species that had moderate to high predictive performance (AUC≥70%). We used the fitted model and the environmental-covariates to predict the probability of each species’ incidence in each grid cell of the study area (‘filling in the blanks’ between the sampling points). The output of this one model is 76 individual and continuous species distribution maps, which we combined to carry out three landscape analyses. First, we counted the number of species predicted to be present (probability of presence≥50%) in each grid square to produce a species richness map. Second, we carried out a dimension-reduction analysis, also known as ordination, using the t-distributed stochastic neighbour embedding (T-SNE) method [53,54] to summarize species compositional change across the landscape. Pixels that have similar species compositions receive similar T-SNE values, which can be visualized. Third, we calculated Baisero *et al.*’s [55] site-irreplaceability index for every pixel. This index is the probability that loss of that pixel would prevent achieving the conservation target for at least one of the 76 species, where the conservation target is set to be 50% of the species’ total incidence.

Finally, we carried out *post hoc* analyses by plotting site irreplaceability, composition (T-SNE), and species richness against elevation, old-growth structural index [56] and inside/outside HJA.

## 3. Results

### (a) Model inputs

#### (i) DNA/taxonomic data

The 121 samples from July 2018 were sequenced to a mean depth of 29.0 million read-pairs 150 bp (median 28.9 M, range 20.8–47.1 M). Of the 190 OTUs used in our JSDM, 183 were assigned to Insecta, and seven to Arachnida (figure 1*b*). All OTUs could be assigned to order level, 178 to family level, 131 to genus level and 66 to species level (figure 1*b*; electronic supplementary material, figure S4).

### (b) Statistical analyses

#### (i) Model performance and xAI

Across all species together, the final JSDM model achieves median and mean explanatory-performance values of AUC=0.86 and 0.86, respectively, where the AUC metric equals 1 for a model with 100% correct predictions and 0 for 100% incorrect predictions. The model’s median and mean predictive AUC (i.e. on the test data) are 0.67 and 0.67 (electronic supplementary material, figure S2*a*). Predictive AUC is a measure of model generality, and the fact that explanatory AUCs are greater than predictive AUCs demonstrates how fitting a model to a particular dataset results in a degree of overfitting. Per species, mean AUC values range from 0 (fail completely) to 1 (predict perfectly), and this variation was not explained by species’ taxonomic family or prevalence (per cent presence in sampling points).

Mean predictive AUC value does not increase with OTU abundance (as measured by incidence), and variability in predictive AUC values is only weakly higher in low-incidence OTUs (electronic supplementary material, figure S12), especially for the OTUs with high mean predictive AUCs (i.e. those used to map species richness, composition and site irreplaceability).

Out of 29 environmental covariates, 18 (electronic supplementary material, table S1) were the most important for at least one species (electronic supplementary material, figure S2*b*). Elevation and TRI were the most important covariates for the most species. Eleven environmental covariates were the most important for at least one species in terms of interaction effects of the variables, with elevation and TRI again being the most important (electronic supplementary material, figure S8).

#### (ii) Prediction and visualization of species distributions

**Figure 2.**
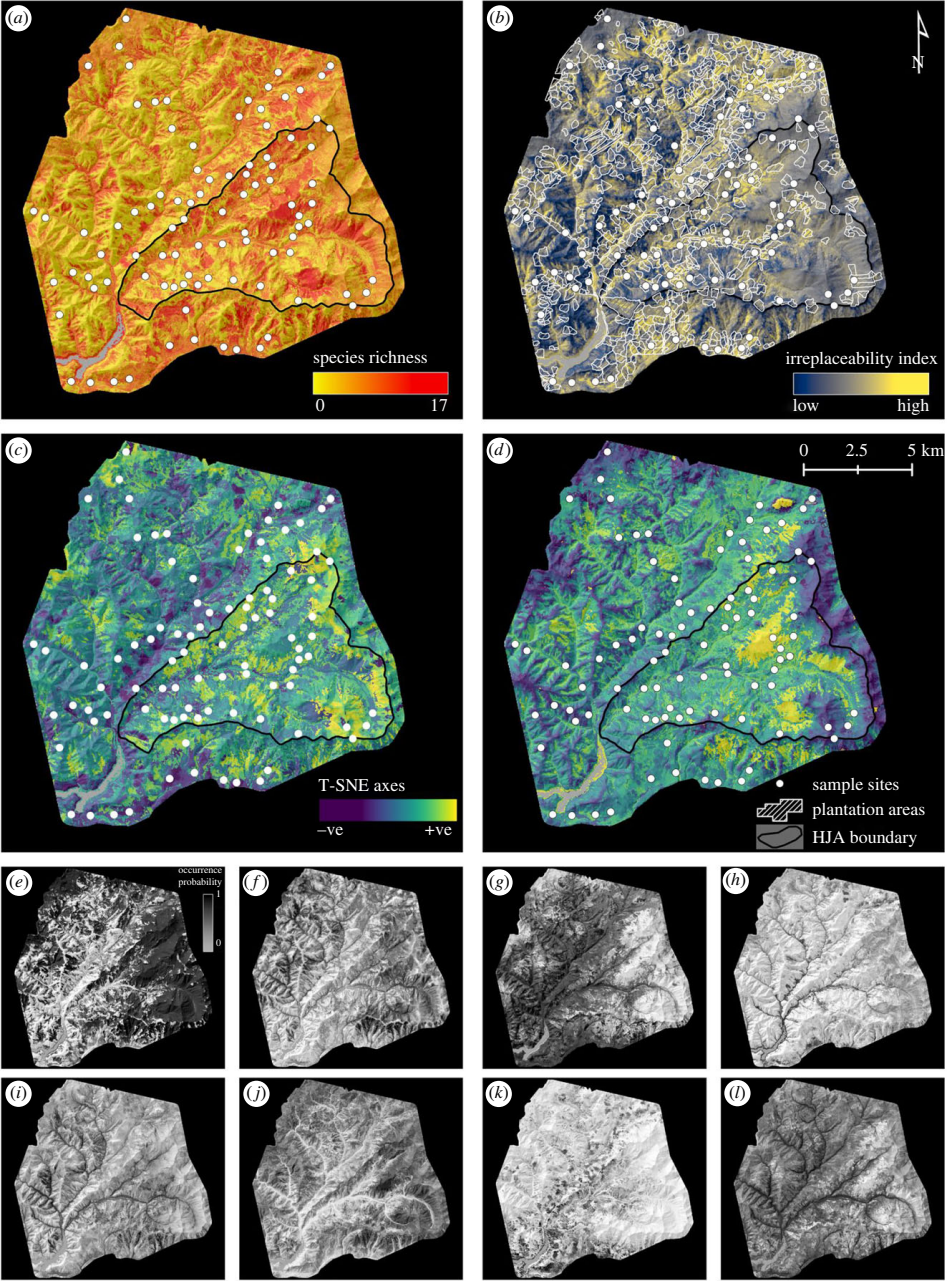
JSDM-interpolated spatial variation in species richness, irreplaceability, and composition, plus examples of individual species distributions. (*a*) Species richness. (*b*) Site beta irreplaceability, showing areas of forest plantation. (*c*,*d*) T-SNE axes 1 and 2. White circles indicate sampling points, white polygons indicate plantation areas (i.e. a record of logging in the last 100 years), and the black-line-bordered triangular area delimits the H.J. Andrews Experimental Forest (HJA; figure 1). (*e*–*l*) Selected individual species distributions (all species in the electronic supplementary material, figure S9), with BOLD ID, predictive AUC and prevalence. (*e*) Rhagionidae gen. sp. (BOLD: ACX1094, AUC: 0.91, prev: 0.64). (*f*) *Plagodis pulveraria* (BOLD: AAA6013, AUC: 0.81, prev: 0.23). (*g*) *Phaonia* sp. (BOLD: ACI3443, AUC: 0.80, prev: 0.65). (*h*) *Melanostoma mellinum* (BOLD: AAB2866, AUC: 0.90, prev: 0.11). (*i*) *Helina* sp. (BOLD: ACE8833, AUC: 0.73, prev: 0.23). (*j*) *Bombus sitkensis* (BOLD: AAI4757, AUC: 0.98, prev: 0.23). (*k*) *Blastobasis glandulella* (Bold: AAG8588, AUC: 0.86, prev: 0.18). (*l*) *Gamepenthes* sp. (BOLD: ACI5218, AUC: 0.77, prev: 0.57).

Finally, we reduced the dataset to the 76 species with individual predictive AUCs ≥ 0.7 (mean = 0.834), and for each, we generated individual distribution maps across the study area, which differ in amount and distribution of the areas with high predicted habitat suitability (figure 2*e*–*l*; electronic supplementary material, figure S9). We then combined the maps to estimate the fine-scale spatial distributions of species richness, community composition and site irreplaceability across the study area (figure 2). Site irreplaceability, which is a core concept in systematic conservation planning, ranks each site by its importance to the ‘efficient achievement of conservation objectives’ [57]. In practice, high-irreplaceability sites tend to house many species with small ranges and/or species with large ranges that we wish to conserve a large fraction of, such as endangered species.

**Figure 3.**
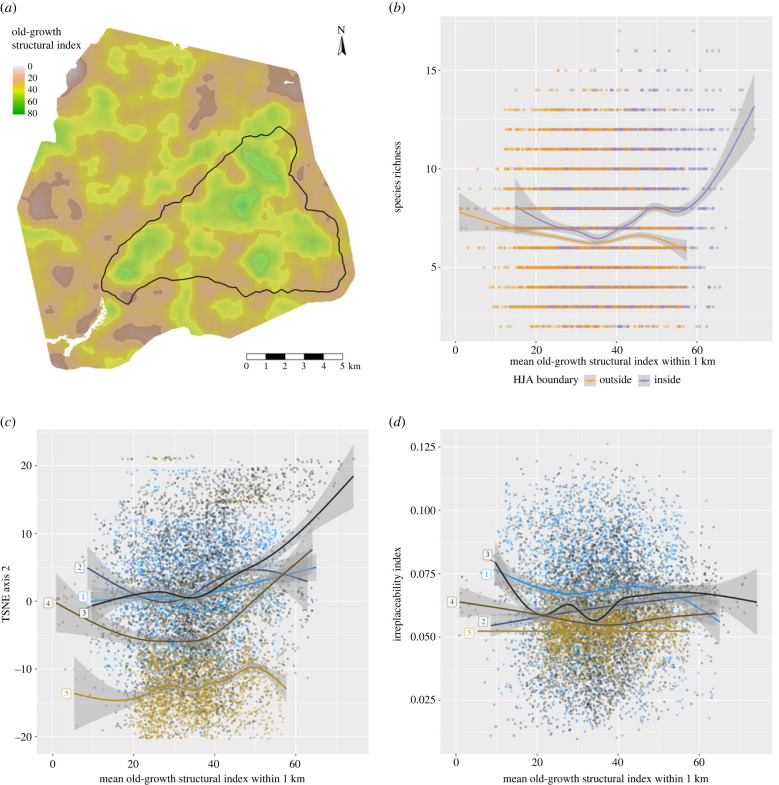
*Post hoc* analysis of species richness, composition and irreplaceability patterns in figure 2, in relation to an old-growth structural index (OGSI) map, from Davis *et al.* [56]. (*a*) Smoothed OGSI, showing principal patches of old-growth forest inside and outside the H.J. Andrews Experimental Forest (HJA; black-line-bordered triangular area). The HJA has the largest patches of old-growth forest. (*b*) Species richness increases in the parts of the HJA with the highest OGSI values (compare with figure 2*a*). (*c*) Species compositions in the largest old-growth patches, which are at elevation bands 3 and 4, are distinct from the rest of the landscape (compare with figure 2*d*). (*d*) Irreplaceability shows no relationship with OGSI at any elevation (compare with figure 2*b*). Elevation bands (blue to brown colour gradient) 1, 380−620; 2, >620−865; 3, >865−1115; 4, >1115−1365; 5, >1365−1615 m above sea level. Splines fit using mgcv [58].

Greater species richness was predicted for areas without recent logging, especially within the northeast and southeast sectors of the HJA, on west-facing slopes, and in the south of the study area (figure 2*a*). A *post hoc* analysis found a nonlinear increase in species richness in the largest patches of old-growth forest, which are inside the HJA (figure 3*a*,*b*).

T-SNE ordination reveals spatial patterning in species composition (figure 2*c*,*d*). T-SNE-1 is clearly correlated with elevation (compare figures 1*a* and 3*c*), whereas T-SNE-2 (like species richness) appears to be correlated with the extent of surrounding old-growth forest, but only at middle elevations (figure 3*c*). Finally, site irreplaceability clearly follows stream courses, which are mostly at low elevations (figure 2*b*) and cover a small portion of the total landscape. As a result, *post hoc* analysis also shows that irreplaceability decreases with elevation but finds no relationship between irreplaceability and surrounding old-growth forest (figure 3*d*).

## 4. Discussion

We combined *in silico* barcode-mapping data derived from 121 arthropod bulk samples in 89 sampling points spread over a 225 km^2^ working and primary forest with 29 environmental covariates (electronic supplementary material, figure S5) from Landsat, LiDAR and other layers that covered information on forest structure, vegetation condition, topography and anthropogenic impact. We used a JSDM with a DNN to predict the fine-scale spatial distributions of 76 Insecta and Arachnida species with a high degree of estimated predictive performance (all individual predictive AUCs > 0.7, mean = 0.834; electronic supplementary material, figure S2*a*). The model made good use of the 29 environmental covariates, with 18 of them being the most important for at least one species (electronic supplementary material, figure S2*b*), with elevation and TRI most important covariates for the most species. These two covariates were also the most frequently most important in terms of their interactions with other covariates (electronic supplementary material, figure S8).

By interpolating to create continuous species distribution maps and combining them, we created *granular* maps of arthropod biodiversity metrics: species richness, community composition and site irreplaceability (figure 2). We observed *post hoc* that species richness is higher and that species composition is distinct in the largest patches of old-growth forest (figure 3*b*,*c*), but not exclusively so. Irreplaceability, as we have defined it here using Baisero *et al.*’s [55] formulation, which does not take connectivity or ecosystem functions into account, is highest along stream courses (figure 3*d*), which are dominated by species with high occurrence probabilities covering a small area (electronic supplementary material, figure S9). Irreplaceability is not higher in old-growth forest, given that old-growth is not a rare habitat in our study area. We consider the patterns observed in figure 3 to be hypotheses for future testing, and thus we do not calculate statistical significance values.

A biodiversity map is more *understandable* than is an analysis of data points and can be compared directly with land-use maps. In principle, these datasets and products can also be *timely*, given that the creation of DNA-based datasets can be outsourced to commercial laboratories in some countries with turnaround times measured in weeks. Information *quality* can be assessed via prediction performance (electronic supplementary material, figure S2*a*), and even *trustworthiness* can be assessed via a combination of proof-of-work GPS surveyor tracking and independent re-sampling, given that sampling is standardized [30].

In summary, we show how to generate information on arthropod spatial distributions with a high-enough resolution to make it useful and understandable for local management while also being efficient and standardized enough to scale up to thousands of square kilometres. However, as shown by the many species with low predictive AUCs (electronic supplementary material, figure S2*a*), future work will be needed to improve how error is accounted for when generating model outputs [30,32], and we discuss methods for doing this in the electronic supplementary material: Caveats. We conclude by briefly reviewing potential applications of this approach.

### (a) Potential applications of efficient, fine-scale and large-scale species distribution mapping

This study demonstrates how the major steps of species distribution mapping are enjoying major efficiency gains [9,19,24,59]. Large numbers of point samples can be characterized to species resolution via DNA sequencing and/or electronic sensors, large numbers of environmental covariates are available from near- and remote-sensing sources [60], and graphics processing unit-accelerated deep learning algorithms can be used to both accelerate and improve model fitting on these larger datasets [42,61]. Although this study focused on arthropods, a wide range of animal, fungal and plant taxa can be detected using DNA extracted from water, air, invertebrate and soil samples [20,29,36,62–68], with river networks being an especially promising way to scale up sampling over large areas [63,69].

As a result, it is possible to envisage implementing Pollock *et al.*’s [44] vision of using ‘sideways’ species-based biodiversity monitoring to subdivide whole landscapes for ranking by conservation value (see also [38]). One potential benefit would be to interpret remote-sensing imagery in terms of species compositions, thus improving the efficiency of habitat-based offset schemes, such as England’s Biodiversity Net Gain legislation, which has been criticized for undervaluing some habitat types, such as scrubland, that are known to support high insect diversity and abundance [70].

Recent studies have also shown that timely and/or fine-resolution biodiversity distribution data can potentially improve conservation decision-making, over that informed by historical distribution data. Ji *et al*. [64] used 30 000 leeches mass-collected by park rangers to map for the first time the distributions of 86 species of mammals, amphibians, birds and squamates across a 677 km^2^ nature reserve in China, finding that domestic species (cows, goats and sheep) dominated at low elevations, whereas most wildlife species were limited to mid- and high-elevation portions of the reserve. Before this study, no comprehensive survey had taken place since 1985, impeding assessment of the reserve’s effectiveness, which is a general problem in the management of protected areas [71]. Chiaverini *et al.* [72] used camera-trap data to extrapolate the distributions of vertebrate species richness across Borneo and Sumatra and found that high species richness areas did not correlate well with the International Union for Conservation of Nature range maps, which are based on historical distribution data (https://www.iucnredlist.org, accessed 18 April 2022). Finally, Hamilton *et al*. [3] compiled decades of standardized biodiversity inventory data for 2216 species in the continental USA and interpolated to identify areas of unprotected biodiversity importance (using a measure similar to site irreplaceability, i.e. protection-weighted range-size rarity). Because the resulting maps were *granular* (990 m), Hamilton *et al*. [3] were able to compare species distributions with land tenure data, including protected areas, and found large concentrations of unprotected species in areas not previously flagged in continental- and regional-scale analyses, in part owing to the inclusion of taxa not normally included in such analyses (especially plants, freshwater invertebrates and pollinators).

### (b) Conclusion

A major difficulty for basic and applied community ecology is the collection of many standardized observations of many species. DNA-based methods provide capacity for collecting data on many species at once, but costs scale with sample number. By contrast, remote-sensing imagery provides continuous-space and near-continuous-time environmental data, but most species are invisible to electronic sensors. By combining the two, we show that it is possible to create a combined spatio(temporal) data product that can be interrogated in the same way as an exhaustive community inventory.

## Data Availability

Raw sequence data are archived at NCBI Short Read Archive BioProject PRJNA869351. All scripts and data tables (from bioinformatic processing to statistical analysis to figure generation) are available from the GitHub respository: https://github.com/chnpenny/HJA_analyses_Kelpie_clean/releases/tag/v1.1.0 and archived at https://zenodo.org/records/8303158 [73]. Supplementary material is available online [74].
